# Evaluation of electric nets as means to sample mosquito vectors host-seeking on humans and primates

**DOI:** 10.1186/s13071-017-2277-3

**Published:** 2017-07-18

**Authors:** Frances Hawkes, Benny Obrain Manin, Sui Han Ng, Stephen J Torr, Chris Drakeley, Tock H Chua, Heather M. Ferguson

**Affiliations:** 1 Agriculture Health & Environment Department, Natural Resources Institute, University of Greenwich, Central Avenue, Chatham, Kent, ME4 4TB UK; 2 Department of Pathobiology and Medical Diagnostics, Faculty of Medicine and Health Sciences, Universiti Malaysia Sabah, Jalan UMS, 88400 Kota Kinabalu, Sabah Malaysia; 3 Department of Vector Biology, Liverpool School of Tropical Medicine, Pembroke Place, Liverpool, L3 5QA UK; 4 Faculty of Infectious and Tropical Diseases, London School of Hygiene and Tropical Medicine, Keppel Street, London, WC1E 7HT UK; 5 Institute of Biodiversity, Animal Health and Comparative Medicine, University of Glasgow, Glasgow, G12 8QQ UK

**Keywords:** *Anopheles balabacensis*, *Plasmodium knowlesi*, Electrocuting traps, Zoonosis, Surveillance, Macaque, Trapping bias

## Abstract

**Background:**

*Plasmodium knowlesi* is found in macaques and is the only major zoonotic malaria to affect humans. Transmission of *P. knowlesi* between people and macaques depends on the host species preferences and feeding behavior of mosquito vectors. However, these behaviours are difficult to measure due to the lack of standardized methods for sampling potential vectors attracted to different host species. This study evaluated electrocuting net traps as a safe, standardised method for sampling *P. knowlesi* vectors attracted to human and macaque hosts.

Field experiments were conducted within a major focus on *P. knowlesi* transmission in Malaysian Borneo to compare the performance of human (HENET) or macaque (MENET) odour-baited electrocuting nets, human landing catches (HLC) and monkey-baited traps (MBT) for sampling mosquitoes. The abundance and diversity of *Anopheles* sampled by different methods were compared over 40 nights, with a focus on the *P. knowlesi* vector *Anopheles balabancensis*.

**Results:**

HLC caught more *An. balabacensis* than any other method (3.6 per night). In contrast, no *An. balabacensis* were collected in MBT collections, which generally performed poorly for all mosquito taxa. *Anopheles* vector species including *An. balabacensis* were sampled in both HENET and MENET collections, but at a mean abundance of less than 1 per night. There was no difference between HENET and MENET in the overall abundance (*P* = 0.05) or proportion (*P* = 0.7) of *An. balabacensis*. The estimated diversity of *Anopheles* species was marginally higher in electrocuting net than HLC collections, and similar in collections made with humans or monkey hosts.

**Conclusions:**

Host-baited electrocuting nets had moderate success for sampling known zoonotic malaria vectors. The primary vector *An. balabacensis* was collected with electrocuting nets baited both with humans and macaques, but at a considerably lower density than the HLC standard. However, electrocuting nets were considerably more successful than monkey-baited traps and representatively characterised anopheline species diversity. Consequently, their use allows inferences about relative mosquito attraction to be meaningfully interpreted while eliminating confounding factors due to trapping method. On this basis, electrocuting net traps should be considered as a useful standardised method for investigating vector contact with humans and wildlife reservoirs.

**Electronic supplementary material:**

The online version of this article (doi:10.1186/s13071-017-2277-3) contains supplementary material, which is available to authorized users.

## Background

Control of vector-borne diseases requires identification of when and where people are exposed to bites from insect vectors, and which vector species are responsible for transmission. Previously, the human landing catch (HLC) has been viewed as the “gold standard” approach for assessing human exposure to mosquito vectors [1]. This procedure requires participants to expose part of their body to mosquitoes, usually the lower leg, and collect all insects that land upon them. As this method gives a direct estimate of the number and infection status of mosquitoes drawn to a human, it is widely used in vector surveillance, research and intervention evaluation. However, this method has a number of limitations. The most serious is that it requires technicians to collect mosquitoes landing on their bare skin and so exposes them to a range of vector-borne diseases, including malaria, dengue, chikungunya virus, filariasis and viral encephalitis, many of which have no prophylactic and/or limited treatment options [2]. The emergence [3] and spread [4] of drug-resistant *P. falciparum* malaria in mainland Asia and the lack of effective prophylaxis for other mosquito-borne pathogens such as dengue and Zika virus make routine use of HLCs particularly problematic in Southeast Asian settings.

Another limitation of HLCs is that, by definition, they can only be performed by humans. In the context of zoonotic disease, there is a need for comparative sampling of mosquitoes biting on both people and other potential animal reservoir species. To date, there are few methods available for comparing mosquito biting rates on people and other animals; and to our knowledge none have been standardised to provide direct comparison with HLC data. Researchers have investigated a range of different sampling methods to collect mosquitoes attracted to animal hosts, including comparisons between HLC and baited net traps for monkeys [5–7]. Although useful for qualitative comparison, inherent differences in the biases and efficiency between sampling methods [8] make it difficult to determine if observed differences between host types are due to their differential attractiveness, or the trapping methods themselves. Accurate and ethically appropriate vector sampling methods for zoonoses thus require trapping approaches that are (i) suitable for use with either humans or other animals as host baits; (ii) limit or eliminate human (and animal) participants’ exposure to vector-borne diseases; and (iii) standardise inherent biases, irrespective of bait animal.

The need to develop standardised methods for measuring mosquito biting has particular relevance for the malaria parasite *Plasmodium knowlesi*. Of all malaria species of public health importance, *P. knowlesi* is the only species with a significant wildlife reservoir, specifically long- and pig-tailed macaques (*Macaca* species) [9]. In 2004, the first large focus of human infection with this zoonotic simian malaria parasite was reported in rural Sarawak, Malaysian Borneo [10]. Since then, this expanding zoonosis has been reported in all Southeast Asian countries with the exception of Laos PDR [11]. Malaysian Borneo is recognized as the epicentre of human cases, with *P. knowlesi* now responsible for the majority of local malaria infections in people [12].

Anopheline mosquitoes of the *Leucosphyrus* group have been implicated as potential vectors in the cross-species transfer of *P. knowlesi* from macaques to humans [11], although other anopheline vectors may be as yet unidentified. Within this group, the vector species responsible for transmission varies geographically. The strongly anthropophilic species *Anopheles dirus* transmits *P. knowlesi* from macaques to humans in Cambodia, Vietnam, China and Thailand [13], while in the west Malaysian state of Pahang, *An. cracens* is considered to be the main vector [7]. However, in east Malaysian states, two vectors have been identified: *An. latens* in Sarawak [14] and *An. balabacensis* in Sabah [15]. The vector species involved in *P. knowlesi* transmission are confirmed to feed on humans, although the degree to which they feed on macaque reservoir species and other wildlife is unclear due to the paucity of available sampling methods. Acquiring such data on relative host preferences of zoonotic malaria vectors is vital for understanding human exposure risk and targeting control measures.

Electrocuting traps may offer a solution to some of the issues associated with traditional mosquito trapping methods [16]. These devices were originally developed to quantify the numbers of tsetse flies attracted to humans and wildlife hosts (warthogs) by placing electrocuting nets in an incomplete ring around the host species [17]. The electrocuting net is effectively invisible to tsetse and hence as they approach the host, tsetse inadvertently collide with it and are either killed or stunned; with the number caught outside and inside indicating their abundance and feeding success. Further variants of these traps have been developed and used to investigate aspects of mosquito behaviour including flight [18] and oviposition behaviour [19]. Early electrocuting traps were often based on placing an electrocuting surface next to the point where host odour was released *via* a pipe (e.g. electric grids [20]), and important subtleties in the behavioural responses of African malaria vectors to odours from humans and livestock cattle have been demonstrated using this approach [21]. Recently developed alternatives work as a barrier placed immediately around a host (e.g. “Mosquito Electrocuting Traps” [22]) and have been shown to be reliable proxies for HLC in some situations [23]. However, the use of such devices to measure mosquito attraction to wildlife has not yet been investigated, nor has their efficacy with more exophilic South East Asian vectors.

Here, we assessed the feasibility of using electrocuting traps as a standardised method to quantify mosquito vector biting rates on humans and macaques, and identify which species have potential to transmit *P. knowlesi* between these hosts. We first tested the suitability of electrocuting nets as an alternative to existing reference trapping methods used for sampling mosquitoes attracted to humans (HLC) and macaques (monkey-baited trap). Secondly, catches from human- and macaque-baited electrocuting nets were compared to determine if and how the mosquito fauna attracted to these different host species vary. Results are discussed with a view to providing new ethically appropriate and scientifically robust methods for vector research and surveillance of zoonotic vector-borne diseases.

## Methods

### Study site

The study was conducted in an area of newly-cleared secondary rainforest outside the village of Tajau Laut (6°57′44.5″N, 116°48′56.3″E) in the Kudat district of Sabah, the northern-most state of Malaysian Borneo. The region has a high prevalence of *P. knowlesi*; 76% of 455 malaria patient samples taken from Kudat District Hospital between 2009 and 2011 were found to be positive for *P. knowlesi* mono-infection by PCR [24]. This village was selected as the study site due to the occurrence of confirmed local cases of human infection with *P. knowlesi* (M. Grigg, pers. comm./Kudat District Hospital) and reports from residents indicating that both long-tailed (*Macaca fascicularis*) and pig-tailed (*Macaca nemestrina*) macaques were frequently seen near the village. Additionally, the presence of *Leucosphyrus* group mosquitoes was confirmed by preliminary HLCs. Experiments were conducted between November 2013 and January 2014, coinciding with the northeast monsoon season of high rainfall and corresponding period of highest local mosquito densities.

### Trapping methods

#### Human landing catch (HLC)

Human landing catches were performed by trained collectors working in pairs. Glass tubes were placed over mosquitoes as they alighted on a collector’s exposed leg, and then the tube was sealed with cotton wool. Collections were carried out between 18:00 and 06:00 h each day. During each hour, mosquitoes were collected for 45 min, followed by a 15 min rest break for the collectors, and stored in bags labelled by hour. Members of the collection team rotated, so that only one exposed their leg during each hour, the other helping to catch the mosquitoes.

#### Monkey-baited trap (MBT)

**Fig. 1:**
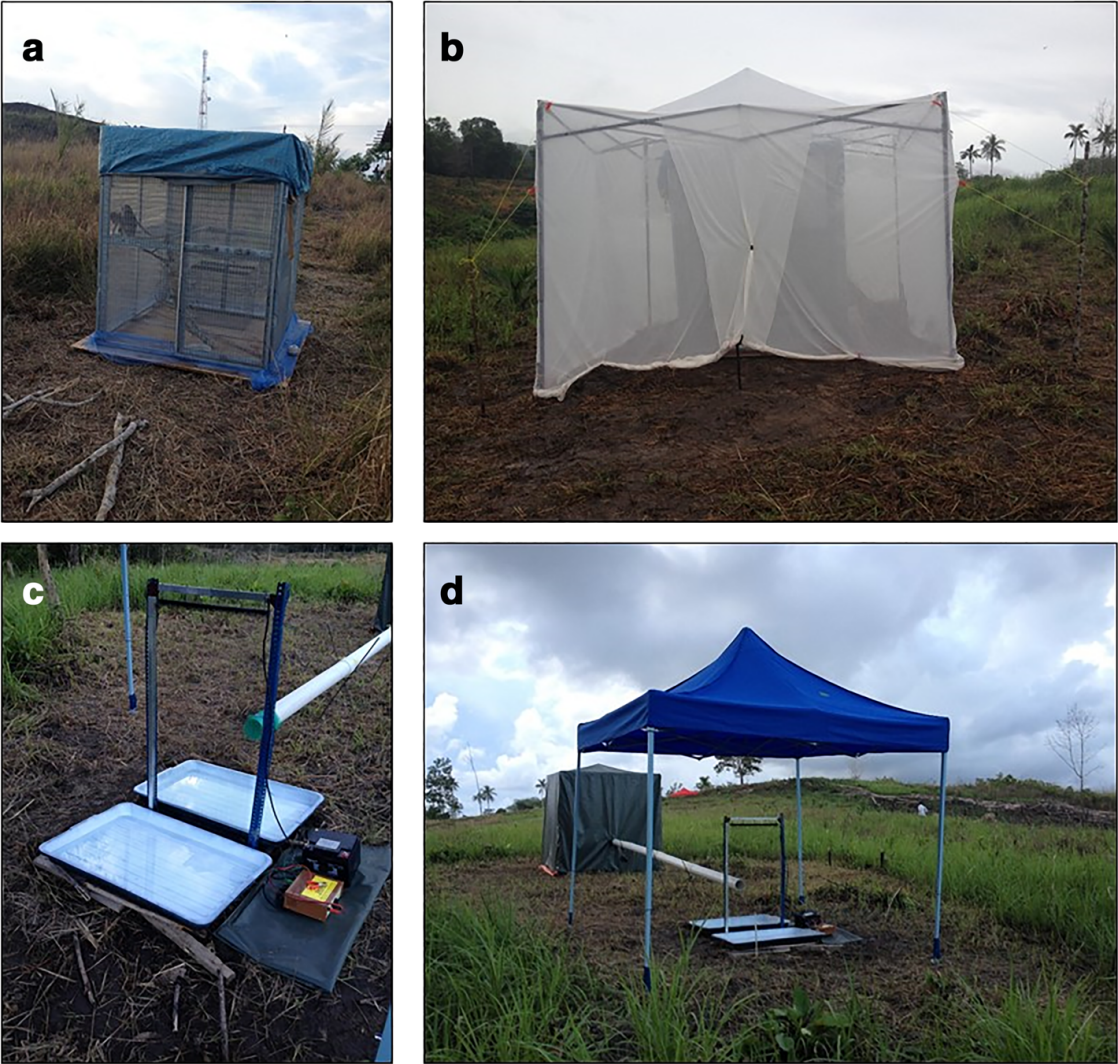
Trapping devices. **a** Ethically compliant cage for two juvenile macaques (see Additional file 1). **b** Monkey-baited trap (MBT): the macaque cage is enclosed in a netted frame, with ~0.4 m of netting rolled up at either end of the net; host-seeking mosquitoes enter the net in search of macaques and are collected from inside the net. **c** Electrocuting net, water collection trays, battery and transformer, with pipe leading from tent. **d** Electrocuting net trap: either two humans (HENET) or two macaques (MENET) are housed in the tent, their odours then vented *via* the tube to an array of charged wires; host-seeking mosquitoes are electrocuted when investigating the host odour and fall into wet collection trays beneath the net

In previous studies of primate malaria vectors, monkey-baited traps have been used as the reference method to sample mosquitoes attracted to macaques [5–7]. We modified this approach slightly as follows: two juvenile long-tailed macaques were placed inside a steel cage measuring 1.8 × 2.0 × 2.0 m and fitted with wire mosquito mesh (2 × 2 mm) to prevent entry of mosquitoes and predators (see Fig. 1a, Ethics approval and Additional file 1 for further details of primate use in research). A metal frame, 2.55 × 2.75 × 2.75 m (i.e. 0.75 m larger than the cage in all dimensions), was erected over the cage and a large, untreated mosquito net was suspended from it, encompassing the cage on all sides. A small opening along the bottom edge at either end of the net was made by rolling up the net from the ground to a height of ~0.4 m (Fig. 1b). Host-seeking mosquitoes were therefore free to enter the net *via* the opening but were prevented from feeding by the cage’s internal mesh, which protected macaques from mosquito bites. This internal protective net has not been previously incorporated into MBT designs and was a requirement of the ethics approvals granted to work with primates. Mosquitoes attempting to exit were either trapped between the cage and outer net, or could leave *via* the 0.4 m floor opening. At the end of each night’s experiments, a collector would enter the mosquito net, close the openings, and collect mosquitoes resting on the inside of the net using glass tubes and cotton wool. These samples therefore represented a whole night’s catch and were not distinguished by hour of collection.

#### Electrocuting nets

Electrocuting nets (1 m high; 0.5 m wide) consisted of a sheet of fine black polyester net sandwiched between two banks of vertical copper wires, 0.2 mm in diameter, 5 mm apart, 8 mm from each side of the net and spray-painted black (Fig. 1c). The net and wires are intended to be invisible to nocturnal flying insects. Alternate wires were earthed or charged by a transformer with a DC input (12 V; 3 A) and an output of 50 kV, pulsing at ~70 Hz. Insects killed or stunned after colliding with the grids were collected in trays placed under either side of the electrocuting net (0.7 m long; 0.5 m wide; 0.15 m deep), each containing ~5 l water and 15 ml dissolved detergent. This design is identical to that developed for tsetse fly research [17], with the exception that vertical wires were spaced 5 mm apart, as opposed to the 8 mm spacing used for tsetse. Previous optimisation experiments in the field showed that a 5 mm spacing was better-suited to local mosquito fauna (data not shown). A marquee erected over electrocuting nets protected them from rain and prevented collection trays from flooding.

Single electrocuting nets were baited with natural host odours from either (i) two humans, or (ii) two macaques (Fig. 1d). Human volunteers were housed inside a tent measuring 2.0 × 2.0 × 2.0 m constructed from plastic-coated canvas; macaques were housed in a cage as described for MBTs, the tent then constructed around this. A coaxial fan (12 V; 0.38 A; maximum airflow ~2000 l/min) drew odour-laden air from the tent down a length of plastic tubing (10 m long, 0.1 m diameter) to a net-covered outlet. The outlet was positioned to release host odour towards the centre of an electrocuting net, with the outlet positioned ~30 cm away from the center of the vertical wire array. As with MBTs, mosquito specimens were collected once at the end of the collection period and retrieved from the collecting trays using fine forceps. The grids themselves were also inspected for any mosquitoes attached to the wires.

### Study design

A comparative evaluation of human-baited (HENET) and macaque-baited (MENET) electrocuting nets against existing trapping methods for these hosts (HLC and MBT, respectively) was carried out between November 2013 and January 2014 in Tajau Laut, Kudat, Sabah. Experiments followed a randomised 4 × 4 Latin square design of traps × sites × nights and took place over 40 nights (10 replicates), sampling within replicates occurring on 4 consecutive nights. Sampling sites were between 100 and 150 m apart and collections were conducted between 18:00 h and 06:00 h. Paired HLC collectors and paired macaques were selected from a pool (8 men; 6 macaques) and rotated randomly throughout the experiment between HLC and HENET, and MBT and MENET, respectively.

### Mosquito identification

After collection, HLC and MBT specimens were returned to the field lab and stored in sealed tubes with silica gel at -15 °C, with samples transferred to -80 °C at the end of each replicate. Wet specimens from electrocuting net collection trays were stored in 70% ethanol. Mosquitoes from each trap were then counted and identified morphologically to genus and then species where possible using a dissecting microscope according to the keys of Sallum et al. [25, 26] for anopheline species, and Rattanarithikul et al. [27] for culicine species.

### *Plasmodium* detection

All 584 *Anopheles* females collected during the study were screened for malaria parasites. Each *Anopheles* individual was cut into two parts (abdomen, and thorax plus head) and the total DNA was extracted from each section using DTAB-CTAB method [28]. Malaria parasites were detected from the specimens using a nested PCR assay as described by Singh et al. [29]. For *Plasmodium* positive specimens, another nested PCR assay was performed to determine the species using nine species-specific primers (Additional file 2: Table S1). Another set of nested PCR assays were performed separately on the same time interval as internal control by targeting the *Anopheles* DNA *cox*2 gene. All three nested PCR assays were performed with 25 μl final volume in the first and second PCR reactions consisting of 5.0 μl of 5× PCR buffer (Promega, Singapore), 0.5 μl of dNTPs (10 mM) mixture (Promega), 3.0 μl of 25 mM MgCl_2_ (Promega), 1.0 μl each of 10 μM forward and reverse primers, 0.3 μl of Tag DNA polymerase (5 U/μl), 2.0 μl of DNA template and sterile dH_2_O up to 25 μl final volume. After completion of the first PCR, 2.0 μl of PCR product was used as DNA template in the second PCR. The PCR conditions were: an initial denaturation at 95 °C for 5 min, followed by 35 cycles of 94 °C for 1 min, annealing for 1 min and 72 °C for 1 min, and a final extension at 72 °C for 5 min. The annealing temperature was set based on the optimum temperature of the primer with range from 50 °C to 66 °C (Additional file 2: Table S1).

### Data analysis

Statistical analyses were performed in R version 3.3.1, with the packages *boot*, *glmmADMB*, *lme4*, *MASS* and *multcomp* [30–35].

#### Mosquito diversity

Three species diversity indices were calculated for each trap type based on all mosquito fauna collected. Species richness (*R*), represented by a count of the total number of different species collected by each trap type, was accompanied by the Gini-Simpson’s Diversity Index (1 − *D*), where:

$$ 1- D=1-\frac{\sum {n}_i\left({n}_i-1\right)}{N\left( N-1\right)} $$

the 95% confidence limit of which is:

$$ \pm 2\sqrt{\frac{{\sum {\left(\frac{n_i}{N}\right)}^2-\left({\sum \left(\frac{n_i}{N}\right)}^2\right)}^2}{N\left( N-1\right)}} $$

Herein, *n*_*i*_ is the abundance of species *i*, and *N* is the total number of individuals in a sample. The relative abundance of different species in each trap was measured using Simpson’s Index of Evenness (*E*), using the formula:

$$ E=\frac{D}{D_{max}} $$

in which *D*_*max*_ is the highest value of *D*, for the given number of species and sample size [36].

#### *Anopheles* abundance

Negative binomial generalized linear mixed models (GLMMs) were used to analyse variation in *Anopheles* species abundance between different collection methods [37]. Trap type was set as a fixed effect and sampling night as a random effect. As ecological data sets often include many ‘0’ counts, models incorporated zero-inflation parameters, but this was found to have a non-significant effect on model fit and was not included as a parameter in the model. Tukey contrasts were used to compare differences in species abundance between trapping methods at α = 0.05.

#### *Anopheles* species composition

Binomial generalized linear models (GLMs) were employed in analysing the proportion of *An. balabacensis* caught in samples from different trapping methods. The proportion of anopheline samples that could not be identified were also analysed to investigate potential differences in sample quality between trapping methods. Tukey contrasts were used to compare differences between trap types.

#### Associations between traps

To assess the agreement in mosquito densities between methods baited with human hosts, Bland-Altman analysis was used [38]. This method provides a graphical representation of potential bias by showing the statistical limits of agreement, and confidence intervals of the mean difference between HLC and HENET, which indicates the magnitude of any systematic differences [39]. The difference between the two methods’ nightly catch was plotted against the mean of their catch. Limits of agreement are considered to be met if 95% of differences fall within ±1.96 standard deviations of the mean difference. Nights on which neither method captured mosquitoes were excluded from analysis, as it is not possible to determine whether the zero count was attributable to lack of sensitivity in the methods, or the genuine absence of mosquitoes. Additional plots of differences between HLC and HENET as a percentage of their mean were also constructed to interrogate variability in differences under different mosquito densities.

#### Human participants

Written informed consent was given by collectors performing HLCs and occupying tents for electrocuting net traps. All human participants were offered doxycycline as an anti-malarial prophylaxis and screened for malaria infection before the study commenced, half way through the study, and again at the end of their enrolment and 2 weeks after the experiment had ended. In the event that a participant showed signs of malaria, rapid assessment and medical treatment was provided at Hospital Kudat. Follow-up monitoring was also offered.

#### Use of non-human primates

The use of non-human primates in this study necessitated compliance with the Animals (Scientific Procedures) Act [40] Code of Practice for the Housing and Care of Animals Used in Scientific Procedures, guidelines set by the National Centre for the Replacement, Refinement and Reductions of Animals in Research [41], and under the authority of the Sabah Wildlife Department. Full details of animal husbandry and safety procedures can be found in Additional file 1.

## Results

Over 40 nights of outdoor trapping, a total of 5679 female mosquitoes were collected (Table 1). Specimens from 38 different species were caught. Culicine mosquitoes represented 90% of the overall catch and comprised 27 species (Table 2) and 584 anophelines representing 11 species made up the remaining 10% (Table 3).

**Table 1:** Summary of mosquitoes caught by each trap type over a 40 night Latin square experiment in Kudat District, Malaysian Borneo

Subfamily	HLC	MBT	HENET	MENET	Total
Anophelinae	403	1	146	34	584
Culicinae	741	369	3407	578	5095
Total	1144	370	3553	612	5679

*Abbreviations*: *HLC* human landing catch, *MBT* monkey-baited trap, *HENET* human-baited electrocuting net, *MENET* monkey-baited electrocuting net

**Table 2:** Relative frequencies of culicine species caught by each trap type, and their medical significance as potential vectors of Japanese encephalitis (JE), chikungunya (CHKV), dengue (DENV) and Getah (GETV) viruses and filarial nematodes

Species	Medical importance^a^	HLC	MBT	HENET	MENET	Total
*Aedes albopictus*	CHKV [49], DENV [50], JE [51]	39	18	3	0	60
*Ae. vexans*	JE [42]	119	25	0	0	144
*Armigeres* species		1	0	0	0	1
*Ar. jugraensis*		0	2	0	0	2
*Ar. malayi*		0	1	0	0	1
*Ar. moultoni*		0	2	0	0	2
*Ar. subabaltus*	JE [52]	1	3	0	0	4
*Ar. kesseli*		2	0	0	0	2
*Coquillettidia crassipes*	Filariasis [53]	7	9	1	0	17
*Cq. ochracea*		3	0	0	0	3
*Culex* species		48	0	1	0	49
*Cx. bitaeniorhycus*	JE [42, 51]	0	1	0	0	1
*Cx. fuscocephala*	JE [43], GETV [44]	7	1	0	0	8
*Cx. gelidus*	JE [43], GETV [44]	39	102	0	1	142
*Cx. malayi*		0	1	0	0	1
*Cx. mimules*		0	1	0	0	1
*Cx. perplexus*		3	2	0	0	5
*Cx. pseudovishnui*	JE [42]	23	27	0	0	50
*Cx. quinquefasciatus*	JE [43], Filariasis [54]	6	21	0	0	27
*Cx. sitiens*	JE [51]	96	13	0	0	109
*Cx. tritaeniorhycus*	JE [43]	35	24	1	0	60
*Cx. vishnui*	JE [42, 51]	135	63	1	1	200
*Cx. whitei*		3	0	0	0	3
*Cx. whitmorei*	JE [44]	2	3	0	0	5
*Mansonia* species		5	0	0	0	5
*M. annulata*	Filariasis [55]	2	0	0	0	2
*M. annulifera*	Filariasis [55]	5	0	0	0	5
*M. dives*	Filariasis [56, 57]	33	2	0	0	35
*M. indiana*	Filariasis [55]	6	0	0	0	6
*M. uniformis*	Filariasis [55]	59	3	0	0	62
Unidentified culicine		62	45	3400	576	4083
Total		741	369	3407	578	5095

*Abbreviations*: *HLC* human landing catch, *MBT* monkey-baited trap, *HENET* human-baited electrocuting net, *MENET* monkey-baited electrocuting net

^a^Reference number

**Table 3:** Frequency of anopheline species caught by each trap type over 40 trapping nights, and their medical significance as potential vectors of human malaria (PHM, primary; SHM, secondary), simian malaria (SM) and the filarial nematodes *Brugia malayi* and *Wuchereria bancrofti*

Species	Medical importance^a^	HLC	MBT	HENET	MENET	Total
*An. balabacensis*	PHM [58], SM [59], *B. malayi* [60], *W.* *bancrofti *[60]	162	0	12	1	175
*An. barbumbrosus* (*s.l.*)		6	0	7	5	18
*An. donaldi*	SHM [61], *B.* *malayi* [59]	3	0	0	0	3
*An. indefinitus*		2	0	0	0	2
*An. kochi*		1	0	0	1	2
*An. latens*	PHM [62], SM [14], *W.* *bancrofti* [60]	19	1	3	0	23
*An. maculatus* (*s.l.*)	PHM [61], *W.* *bancrofti* [61]	14	0	1	0	15
*An. peditaeniatus* (*s.l.*)		1	0	0	0	1
*An. subpictus* (*s.l.*)	SHM [45]	16	0	56	15	87
*An. tessellatus* (*s.l.*)	SHM [61], *W. bancrofti* [61]	179	0	45	8	232
*An. umbrosus* (*s.l.*)		0	0	6	0	6
Unidentified anopheline		0	0	16	4	20
Total		403	1	146	34	584

*Abbreviations*: *HLC* human landing catch, *MBT* monkey-baited trap, *HENET* human-baited electrocuting net, *MENET* monkey-baited electrocuting net

^a^Reference number

Combining across mosquito genera, a total of 31 mosquito species were recorded in HLCs compared to 22 in MBTs. Human and macaque-baited electrocuting nets detected eleven and seven species respectively. Focusing on anophelines, HLCs caught ten different species, HENETs seven and MENETs five. The MBT caught only one anopheline mosquito, *An. latens*. The most dominant *Anopheles* species in this study (out of 584 collected) were *An. tessellatus* (39.7%), *An. balabacensis* (25.9%) and *An. subpictus* (14.9%).

Twenty three of the mosquito species sampled during collections have been implicated as vectors of diseases including malaria, dengue virus and lymphatic filariasis (Tables 2, 3). Of culicince species that could be identified, vector species included *Culex vishnui* (19.8%), *Aedes vexans* (14.2%), *Cx. gelidus* (14.0%), and *Cx. sitiens* (10.8%), which are all known vectors of Japanese encephalitis [42, 43] (Table 2). *Culex gelidus* is also a vector of Getah virus [44]. *Aedes albopictus*, implicated in dengue, chikungunya and Zika virus transmission, was also detected in both human (HLC, HENET) and monkey-baited traps (MENET).

Within the 11 anopheline species detected, six malaria vector species were identified (Table 3), of which *An. balabacensis*, *An. latens* and *An. maculatus* are regarded as significant primary malaria vectors, while *An. donaldi*, *An. subpictus* and *An. tessellatus* are considered secondary vectors with specific geographical areas [25, 26, 45, 46]. With the exception of *An. balabacensis* and *An. subpictus*, all of these malaria vector species are also recognised as vectors of filarial nematodes, including *Brugia malayia* and *Wuchereria bancrofti*. All 584 *Anopheles* specimens underwent PCR analysis to test for the presence of malaria parasites but none were found to be positive. Although only 34 out of 584 captured *Anopheles* mosquitoes were bloodfed, representing less than 0.1% of the total catch, 97% of these came from HLCs highlighting the inherent risk of exposure to mosquito bites during manual collections.

Across anopheline mosquitoes, species richness (Table 4) was greatest in the HLC, followed by the HENET then the MENET, and was lowest in the MBT and no further diversity analyses could be conducted on this method. However, the diversity indices for HLC and electrocuting net collections (both with humans and macaques) were very similar and clustered within 0.06 points of their diversity indices (range 0.64–0.69). The HLC and HENET had identical evenness indices (Table 4), suggesting a similar characterization of overall anopheline community diversity and relative species abundances. The MENET had a slightly higher Evenness index than either HENET or HLC, indicating that although anopheline diversity was lower in the MENET, those species that were attracted to macaque odours were caught in more similar numbers that those attracted to human odours.

**Table 4:** Anopheline species diversity indices by trap type

Index	HLC	MBT	HENET	MENET
Richness, *R*	10	1	7	5
Gini-Simpson’s Diversity Index, 1 − *D* ± 95% CI	0.64 ± 0.002	–	0.69 ± 0.007	0.67 ± 0.001
Simpson’s Index of Evenness *E*	0.04	–	0.04	0.07

*Abbreviations*: *HLC* human landing catch, *MBT* monkey-baited trap, *HENET* human-baited electrocuting net, *MENET* monkey-baited electrocuting net

Quantitative comparisons between traps focus on anopheline mosquitoes and in particular *An. balabacensis* and *An. latens*, due to their implicated role in *P. knowlesi* transmission regionally and elsewhere in Malaysia. Throughout the experiment, the MBT performed poorly for anopheline mosquitoes, only catching one anopheles specimen. Therefore, only limited analysis could be conducted for MBT results.

### *Anopheles* abundance

**Fig. 2:**
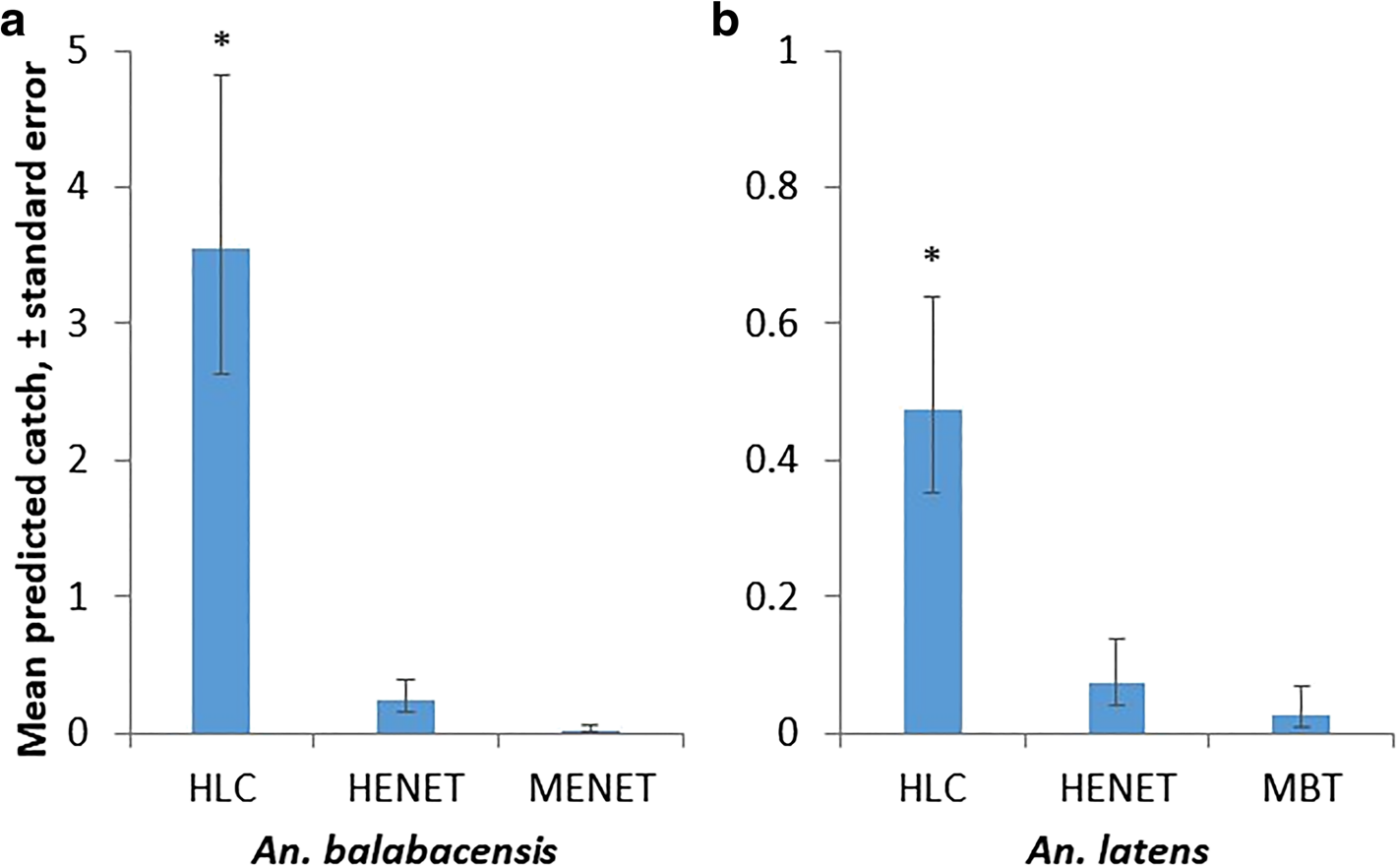
Predicted mean nightly catches (± standard error bars), of potential vectors of *P. knowlesi*; GLMM, *P* < 0.05 (*). **a** *An. balabacensis.* **b** *An. latens*. *Abbreviations*: HLC, human landing catch; MBT, monkey-baited trap; HENET, human-baited electrocuting net; MENET, monkey-baited electrocuting net

The “gold standard” reference HLC method caught the greatest number of anopheline mosquitoes, with total catch descending in the order HLC (*n* = 403), HENET (*n* = 146), MENET (*n* = 34), MBT (*n* = 1). Focussing on *P. knowlesi* vectors, *An. balabacensis* was collected by all methods except MBT; with a range in nightly abundance from 3.6 down to 0.2 per night (Fig. 2a). Human landing catches consistently caught more *An. balabacensis* than any other method (HLC *vs* HENET: *z* = 6.20, *P* < 0.001; MENET: *z* = -4.87, *P* < 0.001; Fig. 2a). However, there was no difference in mean nightly catch of *An. balabacensis*, between electrocuting nets baited with human or macaque odour (*z* = -2.30, *P* = 0.05; Fig. 2a). The number of *An. latens,* another known malaria vector species, also differed significantly according to trap type (*z* = -4.27, *P* < 0.001), with the HLC again catching more than the HENET (*z* = -2.73, *P* < 0.05) and MBT (*z* = -2.77, *P* < 0.01; Fig. 2b). There was no different in *An. latens* nightly abundance between HENET and MENETs (*z* = -0.71, *P* = 0.7; Fig. 2b).

### Representation of malaria vector species

The proportion of *An. balabacensis* within the *Anopheles* mosquitoes collected varied between trap types (% *An. balabacensis*, GLM, *z* = -8.0, *P* < 0.001; Table 3). *Anopheles balabacensis* constituted a higher proportion of the *Anopheles* community in HLC collections (40 ± 2.4%) compared to the HENET (8 ± 2.3%; *z* = 6.3, *P* < 0.001) and MENET collections (3 ± 2.9%; *z* = -3.1, *P <* 0.01). The proportion of *An. balabacensis* did not vary significantly between electrocuting net traps baited with either humans or macaques (*z* = -1.0, *P* = 0.7). *Anopheles latens* constituted 5 ± 1.1% and 2 ± 1.2% of *Anopheles* caught in HLC and HENET samples respectively (no difference between methods, GLM, *z* = 1.3, *P* = 0.4), but this species was absent from MENET collections.

It was possible to identify 97% of anopheline samples to species using morphological features. All unidentified *Anopheles* samples came from electrocuting net traps. From these, 10 ± 2.4% of anophelines in human and 9 ± 4.4% in macaque-baited electrocuting net traps could not be identified to species, with no significant difference in the proportion of unidentifiable specimens between HENET and MENET (GLM, *z* = -0.17, *P* = 0.9). Some diagnostic features such as scales and hairs were found to detach from specimens collected in electrocuting nets, which was the primary reason morphological identification was not possible. In contrast, only 20% of the culicine samples were identifiable on the basis of morphology, mostly from HLC and MBT. Culicine samples from electrocuting net trap samples were stored for longer than anophelines (in 70% ethanol) prior to identification (> 1 year), and this may have resulted in greater loss of delicate diagnostic morphological features.

### Associations between HLC and HENET

**Fig. 3:**
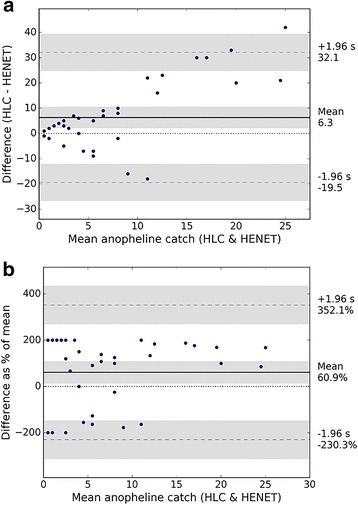
Bland-Altman analysis of total *Anopheles* over 39 nights of catches from human landing catches (HLC) and human-baited electrocuting nets (HENET). The line of equality (dotted line) represents perfect agreement between two methods. Mean difference (solid line) indicates bias from equality, limits of agreement are set at ± 1.96 standard deviations of mean difference (dashed line, s), and both are shown with 95% confidence intervals (shaded areas). **a** Mean *vs* difference. **b** Mean *vs* difference as a percentage of mean

Simple correlation of catches from the HLC “gold standard” method and the alternative HENET method are shown in Additional file 3: Figure S1. In general, Bland-Altman plots indicated a consistency between the number of *Anopheles* caught in HLC and HENET samples (Fig. 3a, b), on the basis of 95% of nightly catches lying within the limits of agreement (Fig. 3a, dashed lines). At lower population densities (under mean catches of ~15 anophelines per night), the difference between nightly HLC and HENET catch is smaller, but increases as density increases. This apparent density-dependence implies that at higher mosquito densities, the HLC captures a greater number of anophelines than the HENET. The observed mean difference between the two methods (Fig. 3a, solid line) indicates that HLC caught an average of six to seven more individual anopheline mosquitoes each night than the HENET. This bias towards HLC is significant; the line of equality at 0 (Fig. 3a, dotted line) represents perfect agreement between the two methods (i.e. no difference in their catch), but falls outside of the observed mean difference and its envelope of 95% confidence intervals.

The density-dependence between HLC and HENET catches can be investigated further by plotting their mean catch against the difference in catch as a percentage of the mean (Fig. 3b). All data points lie well within the limits of agreement at ± 1.96 standard deviations from the mean. The average difference between the two methods is 60.9% of the mean catch (Fig. 3b, solid line), although collections on nights with lower densities of anophelines show deviations of ± 200%. Thus the relationship between the two methods is not consistent over the range of mean catches recorded.

## Discussion

This study represents the first comparison between electrocuting nets and routine methods to sample mosquitoes attracted to human and macaque hosts. A wide range of mosquito species were sampled, encompassing a diverse array of vectors capable of transmitting both human and animal pathogens, including malaria, arboviruses and nematodes. Two recent studies conducted elsewhere in Sabah used the HLC method only to sample potential *P. knowlesi* vectors [47, 48]. Mosquito species composition differed somewhat between these studies, and with results reported here, indicating the huge diversity of human-biting mosquito fauna in this area of Malaysian Borneo and their ecological variability. However, a common finding is the presence of *An. balabacensis*, the locally implicated vector of *P. knowlesi,* both in the present study and previous work [15].

Our key finding is that there is no one optimal sampling method that applies to all mosquito genera. Of the four sampling methods used here, the “gold standard” HLCs had the best performance for anophelines, including key malaria vector species in terms of overall abundance. However, electrocuting nets baited with either humans or macaque monkeys collected the higher number of culicine species, and also show promise for malaria vectors by catching *Anopheles* mosquitoes (~36% relative to HLC). In addition, there was accord between human-baited collection methods, the HLC and HENET, with respect to representative species diversity, which was similar in both. These two methods also shared identical evenness indices. Overall, electrocuting nets provide a standardised, relatively efficient means of sampling host-seeking mosquitoes, on a range of host types, and without exposing collectors to vector-borne diseases. Thus with further optimization to improve the condition of the specimens, they could be a valuable surveillance tool for vector-borne zoonses. One reason the electrocuting nets performed less effectively than HLC could be that the plume of attractive odour released from tents may be reduced relative to the unprotected host, but on the basis of previous studies it is reasonable to assume that host odours were emitted [21]. In our experiment, we standardised traps baited with different animals by number of individuals, always using two humans *versus* two macaques, but standardizing by biomass is an alternative approach, which likely would have yielded quite different results. Our variant of the standard MBT performed worst for anophelines and also caught the lowest number of culicines.

All methods used in this study have limitations. A current weakness of electrocuting nets is that in contrast to other sampling methods around 10% of mosquito samples were damaged to the extent that they could not be identified morphologically. This percentage could perhaps be reduced by retuning the charge delivered by the grid and performing hourly collections from trays. Alternative technology based on the same principles as traditional electrocuting nets is being developed using DC rather than AC power, which facilitates the use of much lower voltages and is associated with higher morphological identification rates in specimens [22, 23]. There is considerable scope to resolve these technical issues and thus provide a sampling method that does not have the ethical concerns associated with HLC.

In our study, monkey-baited traps did not perform particularly well as a sampling tool for anophelines. In previous studies using MBTs, mosquitoes were collected from within the net every 2 h [6] or periodically [7] during the nocturnal sampling period using manual or electrical aspirators. In consultation with Sabah Wildlife Department, neither of these collection methods were deemed appropriate to perform when macaques were in their enclosure, due to the risk to human health of repeated close contact (potential inhalation of viral particles *via* manual aspiration) and animal welfare (excessive noise from electrical aspirators). Therefore, collections were only undertaken at the end of the sampling night, once macaques had been removed. This may have resulted in opportunities for mosquitoes to escape the netting before being collected, accounting for the lower efficiency of MBTs here than reported previously [6, 7]. While comparisons of catches between MBTs are likely to remain a useful tool for understanding more about the ecology of macaque-biting mosquitoes [14], their precise design and operation may have to be modified to be fully compliant with current UK/EU guidelines for research involving non-human primates. Although we did not collect samples hourly during this study for electrocuting net traps, this would be feasible for both human and monkey-baited electrocuting nets, and would provide valuable data that is both comparable to hourly HLCs and important in characterising disease transmission and exposure risk.

Previous *P. knowlesi* vector studies in Southeast Asia have compared MBT catches with HLCs to draw inferences about zoonotic vector host preferences, such as using the ratio of individual mosquitoes from a given species in HLC to that in the MBT. For instance, Jiram et al. [7] calculated the ratio of the *An. cracens* from HLC to MBT to be 1:2.6, suggesting a greater preference for macaque hosts in this vector. However, HLC and MBTs differ greatly in the way in which they sample host-seeking mosquitoes, so potential methodological biases cannot be easily disentangled from real host-specific differences in mosquito attraction. Electrocuting traps, on the other hand, use exactly the same means to sample mosquitoes attracted to host-associated odours, even when the animal producing the odours differs. When all experimental aspects of trapping methodology were standardised here, we found that *An. balabacensis* was caught at a ratio of 12:1 in human-baited to monkey-baited electrocuting nets, although the sample size was relatively low. This raises questions about the host specificity of this vector species; and thus the potential for zoonotic transmission of *P. knowlesi* parasites in Sabah.

## Conclusions

Deciding which vector trapping technology to use in any given context will be a result of balancing technical and practical limitations, ethical issues, financial costs and the nature of required data. The urgent need to replace HLCs with a consistent, exposure-free method is compounded in the context of zoonotic and residual disease transmission settings by the requirement that any alternative method may also need to monitor mosquito activity on other animal hosts. Here we show the potential for electrocuting nets to address both these challenges in the context of zoonotic malaria transmission from wildlife reservoirs to humans. The expense and logistics required to set up electrocuting net traps (< USD 100/unit) makes them suited to long-term monitoring at sentinel sites and use in specific field studies on host-seeking behaviour. These initial costs are offset by the robust comparisons possible between different baits used in the trap, and the protection the method affords to bait animals. Standardised methods, such as electrocuting nets, should be developed further to ensure inferences in vector research are biologically, not methodologically, determined.

## Additional files

Additional file 1: Text. Ethical considerations for the use of non-human primates. (DOCX 16 kb)

Additional file 2: Table S1. Description of primers used for detection of malaria parasite species in *Anopheles* mosquito specimens. (DOCX 47 kb)

Additional file 3: Figure S1. Correlation in catches of anopheline mosquitoes by human landing catch (HLC) and human-baited electrocuting net (HENET). (DOCX 15 kb)

## Abbreviations

GLM: Generalized linear model
GLMM: Generalized linear mixed model
HENET: Human-baited electrocuting net
HLC: Human landing catch
MBT: Monkey-baited trap
MENET: Monkey-baited electrocuting net
PCR: Polymerase chain reaction
PPE: Personal protective equipment
