# Supplementary material for: Evaluation of electric nets as means to sample mosquito vectors host-seeking on humans and primates
Source: Parasit Vectors. 2017 Jul 18;10:338. doi: 10.1186/s13071-017-2277-3 (PMC5516363; doi:10.1186/s13071-017-2277-3)
Supplement: Supplementary file 1 — Text. Ethical considerations for the use of non-human primates. (DOCX 16 kb) [file 13071_2017_2277_MOESM1_ESM.docx]

**Additional file 1. Ethical considerations for the use of non-human primates**

All husbandry and veterinary care for macaques was provided by a team of trained rangers and a veterinarian from Sabah Wildlife Department’s Wildlife Rescue Unit. Prior to involvement in the study, individual macaques underwent a veterinary assessment, including screening for infection with *P. knowlesi*, hepatitis A, B and C, and tuberculosis, and behavioural observations to assess compatibility with other macaques. Their health and welfare was monitored continually by rangers, who were stationed in rotation with the macaques throughout the study; a veterinary assessment of all macaques was conducted every four weeks.

In addition to the diseases listed above, macaques are also commonly infected with B virus (*Macacine herpesvirus* 1, also known as herpes B), although infected animals are often asymptomatic or present only mild symptoms. Human infection with B virus is extremely rare, but results in around 70% mortality in untreated patients, and there is no vaccine available [1]-[2]. To reduce the risk of cross species transfer of this or any other potential zoonosis, both from macaques to humans and *vice versa*, all personnel were required to wear full personal protective equipment (PPE) when working within 1 m of macaques or their cages. This consisted of coveralls, work boots, N100 respirators, goggles, surgical gloves and work gloves.

Following the Animals (Scientific Procedures) Act 1986 Code of Practice for the Housing and Care of Animals Used in Scientific Procedures [3] and guidelines set down by the National Centre for the Replacement, Refinement and Reductions of Animals in Research [4], macaques were held in floor-to-ceiling cages with a minimum height of 1.8 m and minimum enclosure footprint of 2 m^2^ to give each animal a minimum volume of 1.8 m^3^ as stipulated in the Act [4]. Macaques are social animals and so were housed in pairs, following compatibility assessments. Cages were fitted with an elevated platform, to provide roosting space, and enrichment items, including rope, bedding, balls and chew toys [5]. Water was provided freely and diet items hidden around the enclosure and inside toys, as well as being left unpeeled/unshelled to provide stimulation and allow macaques to express natural foraging behaviour [4].

**References**

[1] Cohen JI, Davenport DS, Stewart JA, Deitchman S, Hilliard JK et al. Recommendations for prevention of and therapy for exposure to B virus (*Cercopithecine Herpesvirus* 1). Clin Infec Dis. 2002;35:1191-203.

[2] CDC. B virus. Available at: http://www.cdc.gov/herpesbvirus/index.html (Accessed 18 November 2013).

[3] Animals (Scientific Procedures) Act 1986. Available at: http://www.legislation.gov.uk/ukpga/1986/14/contents (Accessed 31st October 2016).

[4] NC3Rs. Guidelines – Primate accommodation, care and use. London: NC3Rs. 2006.

[5] Waitt CD, Honness PE, Bushmitz M. Creating housing to meet the behavioural needs of Long-tailed macaques. Primate Newsletter. 2008;47(4):1-5.
