# Supplementary material for: Evaluation of electric nets as means to sample mosquito vectors host-seeking on humans and primates
Source: Parasit Vectors. 2017 Jul 18;10:338. doi: 10.1186/s13071-017-2277-3 (PMC5516363; doi:10.1186/s13071-017-2277-3)
Supplement: Supplementary file 2 — Description of primers used for detection of malaria parasite species in Anopheles mosquito specimens. (DOCX 47 kb) [file 13071_2017_2277_MOESM2_ESM.docx]

**Additional file 2: Table S1.** Description of primers used for detection of malaria parasite species in *Anopheles* mosquito specimens

| Target | *Plasmodium* | Primer name | Sequence (5’ - 3’) | Annealing temp. | Reference |
| --- | --- | --- | --- | --- | --- |
| SSU-rRNA | *Plasmodium* genus | rPLU1 | TCAAAGATTAAGCCATGCAAGTGA | 55^o^C | [1] |
|  |  | rPLU5 | CCTGTTGTTGCCTTAAACTCC |  | [1] |
|  | *Plasmodium* genus | rPLU3 | TTTTTATAAGGATAACTACGGAAAAGCTGT | 62^o^C | [1] |
|  |  | rPLU4 | TACCCGTCATAGCCATGTTAGGCCAATACC |  | [1] |
|  | *P. coatneyi* | PctF1 | CGCTTTTAGCTTAAATCCACATAACAGAC | 62^o^C | [2] |
|  |  | PctR1 | GAGTCCTAACCCCGAAGGGAAAGG |  | [2] |
|  | *P. inui* | PinF2 | CGTATCGACTTTGTGGCATTTTTCTAC | 60^o^C | [2] |
|  |  | INAR3 | GCAATCTAAGAGTTTTAACTCCTC |  | [2] |
|  | *P. fieldi* | PfldF1 | GGTCTTTTTTTTGCTTCGGTAATTA | 66^o^C | [2] |
|  |  | PfldR2 | AGGCACTGAAGGAAGCAATCTAAGAGTTTC |  | [2] |
|  | *P. cynomolgi* | CY2F | GATTTGCTAAATTGCGGTCG | 60^o^C | [2] |
|  |  | CY4R | CGGTATGATAAGCCAGGGAAGT |  | [2] |
|  | *P. knowlesi* | PkF1140 | GATTCATCTATTAAAAATTTGCTTC | 50^o^C | [3] |
|  |  | PkR1550 | GAGTTCTAATCTCCGGAGAGAAAAGA |  | [3] |
|  | *P. falciparum* | NewPLFshort | CTATCAGCTTTTGATGTTAG | 53^o^C | [4] |
|  |  | FARshort | GTTCCCCTAGAATAGTTACA |  | [4] |
|  | *P. vivax* | NewPLFshort | CTATCAGCTTTTGATGTTAG | 53^o^C | [4] |
|  |  | VIRshort | AAGGACTTCCAAGCC |  | [4] |
|  | *P. malariae* | NewPLFshort | CTATCAGCTTTTGATGTTAG | 53^o^C | [4] |
|  |  | MARshort | TCCAATTGCCTTCTG |  | [4] |
|  | *P. ovale* | NewPLFshort | CTATCAGCTTTTGATGTTAG | 53^o^C | [4] |
|  |  | OVRshort | AGGAATGCAAAGARCAG |  | [4] |
|  | *Anopheles* |  |  |  |  |
| COII | *Anopheles genus* | COIIF | TCTAATATGGCAGATTAGTGCA | 50^o^C | [5] |
|  |  | X2R | TGATTTAAGAGATCATTACTTGC |  | This study |
|  | *Anopheles genus* | X2F | GGCAGATTAGTGCAATGAATT | 53^o^C | This study |
|  |  | COIIR | ACTTGCTTTCAGTCATCTAATG |  | [5] |

1. Singh B, Bobogare A, Cox-Singh J, Snounou G, Abdullah MS, Rahman HA. A genus- and species-specific nested polymerase chain reaction malaria detection assay for epidemiologic studies. Am J Trop Med Hyg. 1999;60(4):687-692.

2. Lee KS, Divis PCS, Zakaria SK, Matusop A, Julin RA, Conway DJ et al. *Plasmodium knowlesi*: Reservoir hosts and tracking the emergence in humans and macaques. Plos Pathog. 2011;7:4.

3. Imwong M, Tanomsing N, Pukrittayakamee S, Day, NPJ, White NJ, Snounou G. Spurious amplification of a *Plasmodium vivax* small-subunit RNA gene by use of primers currently used to detect *P. knowlesi.* J Clin Microbiol. 2009;47(12):4173-4175.

4. Ta TH, Hisam S, Lanza M, Jiram AI, Ismail NP, Rubio JM. First case of a naturally acquired human infection with *Plasmodium cynomolgi*. Malaria J. 2014;13:68.

5. Yang MN, YJ Ma, Wu J. Mitochondrial genetic differentiation across populations of the malaria vector *Anopheles lesteri* from China (Diptera: Culicidae)*.* Malaria J. 2011;10:216.
