# Supplementary material for: Evaluation of electric nets as means to sample mosquito vectors host-seeking on humans and primates
Source: Parasit Vectors. 2017 Jul 18;10:338. doi: 10.1186/s13071-017-2277-3 (PMC5516363; doi:10.1186/s13071-017-2277-3)
Supplement: Supplementary file 3 — Correlation in catches of anopheline mosquitoes by human landing catch (HLC) and human-baited electrocuting net (HENET). (DOCX 15 kb) [file 13071_2017_2277_MOESM3_ESM.docx]

**Additional file 3: Figure S1.** Correlation in catches of anopheline mosquitoes by human landing catch (HLC) and human-baited electrocuting net (HENET)
